# Learning or leaving? An international qualitative study of factors affecting the resilience of female family doctors

**DOI:** 10.3399/bjgpopen20X101017

**Published:** 2020-03-18

**Authors:** Alice Shiner, Jessica Watson, Noemi Doohan, Amanda Howe

**Affiliations:** 1 GP Research Fellow, Norwich Medical School, University of East Anglia, Norwich, UK; 2 NIHR Doctoral Research Fellow, Centre for Academic Primary Care, Bristol Medical School, University of Bristol, Bristol, UK; 3 Assistant Program Director, Scripps Chula Vista Family Medicine Residency Program, Department of Family and Community Medicine, San Diego, California, US; 4 Professor of Primary Care, Norwich Medical School, University of East Anglia, Norwich, UK

**Keywords:** family practice, primary health care, women, resilience, transition, health workforce

## Abstract

**​Background:**

Many countries have insufficient numbers of family doctors, and more females than males leave the workforce at a younger age or have difficulty sustaining careers. Understanding the differing attitudes, pressures, and perceptions between genders toward their medical occupation is important to minimise workforce attrition.

**​Aim:**

To explore factors influencing the resilience of female family doctors during lifecycle transitions.

**​Design & setting:**

International qualitative study with female family doctors from all world regions.

**​Method:**

Twenty semi-structured online Skype interviews, followed by three focus groups to develop recommendations. Data were transcribed and analysed using applied framework analysis.

**​Results:**

Interview participants described a complex interface between competing demands, expectations of their gender, and internalised expectations of themselves. Systemic barriers, such as lack of flexible working, excessive workload, and the cumulative impacts of unrealistic expectations impaired the ability to fully contribute in the workplace. At the individual level, resilience related to: the ability to make choices; previous experiences that had encouraged self-confidence; effective engagement to obtain support; and the ability to handle negative experiences. External support, such as strong personal networks, and an adaptive work setting and organisation or system maximised interviewees’ professional contributions.

**​Conclusion:**

On an international scale, female family doctors experience similar pressures from competing demands during lifecycle transitions; some of which relate to expectations of the female's ’role’ in society, particularly around the additional personal pressures of caring commitments. Such situations could be predicted, planned for, and mitigated with explicit support mechanisms and availability of workplace choices. Healthcare organisations and systems around the world should recognise this need and implement recommendations to help reduce workforce losses. These findings are likely to be of interest to all health professional staff of any gender.

## How this fits in

To the authors’ knowledge, this study is the first to explore female family doctors’ perceptions of factors affecting their resilience during personal and professional transitions. The findings expose, to conscious analysis, the private battles of female family doctors as they attempt to deliver on their personal and professional opportunities. The present study shows the huge importance of organisations having emotional intelligence and the crucial need to support people at transitions in their lives, especially if these are unexpected, have a negative cause, and happen early in careers. Through this study, globally relevant lessons can be taken about how to maintain female family doctors’ workforce contribution.

## Introduction

The health workforce is a precious resource, especially when it is small relative to the population it serves. The World Health Organization estimates a shortage of 18 million health workers worldwide;^1^ therefore, it is concerning to find that females entering the medical workforce appear to be leaving at a young age or are not able to sustain their careers as intended.^2^ This may be due to issues for females in the medical workforce set out in recent *Lancet* commentary, which indicates poor career progression, worse pay and conditions, and persistence of the ‘glass ceiling’ at professional leadership levels.^3,4^

Gendered expectations of females as caregivers can influence their working patterns and careers,^5^ and also impact on the welfare of their families and the healthcare system they work within.^6^ Structural factors, such as availability of flexible working or legal protection during periods of ill health or childrearing, may make a difference between retaining or leaving a job, as may the attitudes and capacity of colleagues.^7^

Much of the available literature comes from high-income countries, but as the female medical workforce grows, similar impacts on gender in medical careers are emerging in global settings.^8^ When looking at factors influencing workforce retention, constructs of psychological resilience and vulnerability^9^ — as well as organisational,^10^ cultural,^11^ and systemic factors —^12^ are important. Doctors must establish a professional identity,^13^ and develop technical and emotional independent practice in a situation of high responsibility and demand.^14^ In addition, they have both planned and unplanned transitions throughout their adult lives.^15^ There is evidence that transitions are associated with people leaving the workforce; this appears to be more common for female doctors.^16^

Gender has an impact on every level of the health workforce,^17^ where the majority of staff are female. The diversity of systems with different legislations^18^ and the provision of care by the private sector^19^ adds an additional dynamic. Family medicine is a substantial part of the medical workforce in many countries. In Europe^20^ and many other world regions it is perceived as being a more ‘family friendly’ specialty; however, as most family doctors work in small teams, in settings with a less well-developed organisational structure, there tend to be fewer formal and structural solutions for personal situations. As there is a worldwide push to strengthen primary care to deliver effective universal health coverage,^21^ the discipline of family medicine is becoming more developed at a global level,^22^ which makes learning about the experiences of this sector particularly relevant.

In this context, one important question is what makes an individual leave or persist within a working environment when there are challenges? This study aimed to address this question and to examine whether there are factors where intervention might maximise career engagement and minimise workforce loss. The present study focuses on lifecycle transitions, seeking to explore factors influencing the resilience of female family doctors (including the ability to make adversity meaningful, thrive in spite of adversity,^23^ and remain productive in the workplace). It evolved from the interests, observations, and lived experiences of the authors and their engagement with international networks in the World Organization of Family Doctors (WONCA), specifically the WONCA Working Party for Women and Family Medicine, which has a programme of research and collaboration on the impacts of gender in family medicine.^24^

## Method

This was a qualitative study utilising semi-structured interviews conducted online via Skype, followed by in-person focus groups to validate themes and develop recommendations. The study grew out of an action research cycle, beginning with a series of scoping workshops on the topic of female family doctors and career resilience at WONCA events in the seven world regions. This led to an initial framework of key issues, which were then refined into the interview protocol.

The overall research question was, *’What do female family doctors around the world believe influences their professional resilience during transitional life events?*’ The purpose was to deepen understanding and make recommendations on what could make a difference in maximising the careers and lives of female family doctors.

### ​Recruitment and sampling

The study was advertised to potential participants via electronic mailing lists and social media networks of WONCA across all seven world regions (Africa, Asia Pacific, East Mediterranean, Europe, Iberoamericana-CIMF [Confederación Iberoamericana de Medicina Familiar], North America, South Asia).

Inclusion criteria were: (1) sufficiently proficient in English to conduct an in-depth qualitative interview; (2) defines main speciality practice as a family doctor (though could be on a service break); (3) female; and (4) have access to an internet connection with Skype software.

Purposive sampling was used to select participants with a range of demographic characteristics within and between world regions.

### ​Interviews

Interviews were conducted by three authors between 6 March–3 August 2017 using Skype. Interviews were audio recorded with an encrypted device and lasted between 24–61 minutes (mean 43 minutes, median 53 minutes). An experienced research assistant transcribed the recordings. Participants had the opportunity to check and correct the transcript.

The interviews were semi-structured with non-directional questions. The topic guide (see Supplementary Box S1) covered a range of issues developed from the scoping workshops. To ensure that it was understandable and culturally appropriate for doctors from a range of backgrounds, it was piloted and refined through review by the study’s International Advisory Group (seven senior female academics active in WONCA from all world regions). New questions evolved during the study to explore emerging themes. Interviews were continued until themes were repeated and no new major areas were emerging (data saturation). Known core dimensions of resilience (’5 Cs and an M’: confidence, commitment, coordination, control, composure, and making adversity meaningful^23^) were found to have explanatory value in the early stages of the study, and these dimensions were used in the framework for analysis for much of the personal data in the interviews.

### ​Focus groups

Findings from the interviews were iterated into three focus groups at WONCA regional conferences during 2018 (in Kuwait, Colombia, and Poland). Participants were recruited via WONCA networks. Inclusion criteria were that the participant: (1) was a practising family doctor; (2) was able to take part in a focus group discussion conducted in English; and (3) self- identified as a leader in the profession (for example, leaders of academic departments or family medicine services). All focus groups were run and convened by the same author using the same prompt sheet (see Supplementary Box S2) to encourage participants to note the key findings of the project and how these might be addressed. The sessions lasted 30–60 minutes. The focus group in Colombia was conducted partly in Spanish and had a broader attendance; the convening author kept field notes and made a summary of key points, but full transcription was not possible. The other two groups were audio-recorded and transcribed and used as primary data, with the Colombia data used only to add weighting to primary coding from the other two groups.

### ​Analysis

Analysis was carried out using applied framework analysis,^25^ using a framework developed from the scoping workshops. This framework iteratively developed during the process of analysis. Dedoose (an online cross-platform software application for analysing qualitative research) was used to allow all of the team to access and analyse the data. Analysis took place concurrently with interview data collection to allow testing of emerging themes in subsequent interviews.

Transcripts were analysed by two authors, with one author reviewing coding to enhance validity. Initial open codes were grouped into linked axial codes, which were then grouped into higher-level theoretical codes that described particular domains of participants’ experiences. The wider domains related to individual, organisational, and systemic aspects of participants’ lives alongside descriptions of the lived experience. Within each domain, some codes were more gendered than others (for example, ‘gender roles in family’ was a more gendered code than ‘workplace demands’, although both related to the organisational domain of participants’ lives). See Supplementary Figure S1 for the final framework. Disagreement about coding decisions and the development of higher-level codes were discussed during meetings with all team members to improve the development of insights and new meaning from the data. Focus group data were analysed to create key themes around recommendations. These themes were tested against the original framework, with discussion between team members allowing for the development and triangulation of emergent concepts and theories.

## Results

### ​Participants

In total, 38 female family doctors responded to the advertisement for interviews; 20 were purposively selected as participants based on age, location, and years of experience as a family doctor. One participant withdrew; their data were not included in the analysis. See Table 1 for interview participant characteristics and Table 2 for the focus group participant characteristics.

**Table 1.** Interview participant characteristics

**WONCA world region**	**Participants, *n***	**Age range, years**	**Range in practice, years^a^**
Asia Pacific	3	37–52	11–25
Africa	2	42–54	10–21
North America	4	42–61	3–24
Iberoamericana-CIMF	3	31–60	3–31
Europe	3	32–44	1–14
South Asia	2	43–55	14–15
East Mediterranean	2	47–57	18–28

^a^One participant was working part-time, three were on career breaks, and all others worked full-time. Four participants worked clinically as a family doctor, the other 15 combined clinical work with work as an educator in academia or healthcare management. CIMF = Confederación Iberoamericana de Medicina Familiar. WONCA = World Organization of Family Doctors.

**Table 2.** Focus group participant characteristics

**Country**	**Participants, *n***	**Gender**	**Countries represented, *n***	**Other characteristics**
Kuwait	8	M2:F6	8	6 family doctors, 2 other specialities; all ‘leaders’ in some form
Colombia	14	M3:F11	3	All family doctors; varying range of experience, including residents
Poland	8	F8	6	All family doctors; all but one on WONCA regional councils

F = Female. M = Male. WONCA = World Organization of Family Doctors.

### ​Findings

Transitions could be characterised as personal and professional; planned and unplanned; and historic or current. Examples included: parenthood; moves between countries; moves caused by family needs; and career changes. Two major themes emerged from the analysis: the challenge of competing demands and the need for support at different levels to bolster inherent resilience.

### ​Lived experience and ‘competing demands’

Much of the data described the ‘lived experience’ of transition. Competing demands and pressures from work and home life were often most challenging during lifecycle transitions. Although it was rare for participants to describe overt gender discrimination, cultural expectations of females were commonly described and often internalised. Such expectations added to the pressure of competing demands: *‘I always felt that I had to balance my career and I had to be the perfect mum at home and perfect wife and perfect daughter’* (Participant [P]37). This could lead to feelings of guilt or ‘failure’: ‘*I’ve always had that guilt of am I not giving enough?’* (P3). In the few examples where a male partner had taken a lead role in family responsibilities, the language used in describing these examples showed that this was seen as exceptional and optional: ‘... *he doesn’t stop me ...’ (P16); ‘... he didn’t actively prevent me ... ’* (P39).

The challenge of competing demands was amplified by a culture within medicine that prized hard work and self-sacrifice as a ‘... *badge of honour ...’* (P3), and some felt that as a female doctor they needed to do even more to be successful: *‘I think as a woman* [in medicine] *I always feel that we have to work harder to be taken seriously’* (P37).

### ​Resilience: ‘can be inherent, but needs support’

A variety of individual, organisational, and systemic factors influenced the resilience of participants. At an individual level, the key characteristics of resilient people (confidence, control, composure, coordination, commitment, and making adversity meaningful)^23^ were reflected in some participants’ accounts*: ‘I had friends who said to me they understood why I was leaving and they seemed to be supportive. But I was not looking for permission or validation from them as I felt strongly that this was a change I needed to make.’* (P3). Completing, learning from, and having an opportunity to reflect on transitions seemed to help: ‘... *a little break helped me think what I really want to do ...’* (P26). Participants displaying resilient characteristics also appeared more able to access support and make use of organisational factors to assist with transitions; however, the context of the transition mattered. Autonomy and having the opportunity to make choices had a positive impact; if this was taken away, it could be difficult: ‘... *it was really hard especially ... when the decision kind of had been made for you, that was really difficult ...’* (P2).

Organisational factors were important in both the personal and professional domains of participants’ lives, particularly the availability of a network for psychological and/or practical support (family and/or friends in the personal domain and colleagues professionally): *‘I’ve got amazing people that support me and then I can bounce ideas off and I think that does add to my resilience cos like I have such an amazing network of people who I work together with ...*’ (P2). Childcare and running the household was generally considered a female’s responsibility; almost all participants with children needed flexible help at home (employed or a relative) to be able to work: *‘... if a patient is sick and I have to make two house calls I don’t know what time I will be home so definitely day care and my mother helps a lot ...’* (P33). In the workplace, the presence of understanding colleagues who provided peer support often helped interviewees to manage high levels of pressure and demand, and, while not available to all, the support of a named mentor (male or female) was helpful. Notably, participants did not require constant support, more the ability to seek the right support at a point in time: *‘I’ve got mentors in different aspects of my life … like, different people for different things ...*’ (P2).

There was widespread variability in the availability of organisational support in the form of maternity or caregivers’ leave and part-time or flexible working. Some participants had no such options while others had substantive periods of paid maternity leave or could make choices about their working hours. In systems with little support, females were making choices that involved financial or career sacrifice: ‘... *well yes women do take time off but it is at their own expense ...’* (P14); while those with access to support, such as flexible working, enjoyed the control and felt it helped them continue in work: ‘... *so for me it’s very important to work part-time in order to still enjoy what I do ...’* (P29).

In some countries, a lack of recognition of family medicine as a specialty exacerbated issues with poor systemic support, as did inadequate systems resource, with participants struggling as a consequence: *‘I passed like 6 months without a nurse in my team so that was hard to a lot of work to do without any assistance from the other teams that works with me ...’* (P6). Even when legislation existed to improve working conditions, this did not always translate into practice: *‘... they haven’t done anything to make these things happen like all the rules are like they are there on the white board or whatever but we can’t get any benefit out of that ...’* (P37). However, in some cases individuals felt able to effect systemic change: ‘*When we realised we could not make it work, I approached my department and colleagues to make more adjustments to the schedule to help me. My resilience gave me the power, or maybe it was audacity, to approach my colleagues and ask for help ...’* (P3).

### ​Focus groups

The groups considered what could be done for and by individuals (‘micro‘), teams or local organisations (‘meso’), and at systemic or national (‘macro’) levels. Some issues, such as the need to increase knowledge about and recognise the challenges that transitions can present, crossed all three levels. The main themes to arise are detailed in the final column of Table 3, and the recommendations could be classified into five categories of activity (Box 1). A key feature of the focus group data was that it moved the findings on from focusing on individuals to solutions at organisational or systemic levels. This resonated with the interview data, which found that any personal solutions involving individual’s resilient characteristics had a greater chance of operating in a more supportive environment. It also reflects the context of WONCA as a professional network that aims for systemic changes, rather than expecting individuals to solve their own problems.

**Table 3.** A summary of the major challenges to the resilience of female family doctors and possible recommended solutions at the personal, team, and systemic level

**Challenges**	**Examples**	**Recommendations**
**At the personal level**		
Unplanned transitions pose a greater threat to resilience. Control is important.	‘*I suppose it undermined my confidence as well because I felt that well ok this is something that I’d not planned and so I thought people may think I am irresponsible or whatever ...’* (P37)	**1.** **Rehearse possible scenarios** **:** planned life transitions, such as a career shift, getting pregnant, or retiring, are all good examples of transitions where the next step can be rehearsed. Talking to people who have already had similar experiences, exploring implications with significant others, and listening to your own fears and doubts can all be useful. Planning for the unexpected can help when unplanned life transitions arise, for example, illness or sudden loss.
Gendered expectations lead to pressure on females from multiple competing demands, influencing resilience.	*‘There were some nights I was on call on labour and delivery and they would text me pictures of their homework and we would do homework over the phone, while I was watching the monitor of a patient in labour. This was not the way I wanted to be a mum to them, and this was not the way I wanted to be a doctor. I was pulled in two different directions and not doing either job well.’* (P3)	**2** **. Be kind to yourself** **and take a long** **-** **term view** **:** decide on your priorities and make proactive choices to enable you to achieve these. Ask for help and do not put pressure on yourself to be, for example, the ‘perfect doctor’ and the ‘perfect mother’. Take time for yourself and protect your own personal interests.
Individuals need access to personal support networks to build and retain resilience.	*‘So when I speak about my resilience or how I am able to adjust to make it work, I must also speak about my support, my village, my community ...’* (P3)*‘So I’m lucky here that I do have a few very close friends that feel like a family to me actually so I guess where you go you kind of you know you find people I suppose that understand you* ...’ (P8)	**3. Ensure that you access supportive others who have relevant experience and expertise** **:** friends, colleagues, professional support networks, mentors, and your own doctor or occupational health lead can all be useful. Doctors often need to give themselves permission to confide in others and ensure confidentiality, but the literature shows that sharing concerns and allowing emotions to surface are important for successful transitions, particularly if these are unexpected and/or traumatic. Being in a new situation can be isolating; reach out early to find local support or plan regular contacts with previous friends and colleagues via the internet.
**At a team level**		
Isolation is a risk during some transitions, for example, those early in a career, working in more dispersed settings, or returning to work from a period of leave.	*‘I was alone with my two partners who were old and close to leave so they didn’t want to make any innovation in the office and it was very difficult for me because it wasn’t what I wanted ...’* (P26)	**4** **. Create support structures and personnel leads as well as protocols and guidance so that everyone in a team knows how to access these when transitions occur** **:** these may be guided by legal or personnel frameworks and may also be needed when people return to work, as this is often a period of stress and uncertainty.
Lack of formal or informal mentorship can be a barrier to resilience, as can a lack of female role models.	*‘I had good examples from our senior mentors* [consultants] *like they were mums they raised families they raised kids and they all turned out to be good family physicians … and they’re good leaders in our national organisation and they’re such an inspiration for us younger consultants or younger practitioners ...’* (P23)	**5. Offer a contact or mentor for a specific situation** **:** mentorship in a new role or a return to work enables proactive support. Having a named person gives the message that it is acceptable and important to seek help.
Inflexible working hours, insufficient affordable childcare, and poor support for those working LTFT may lead to females leaving the workforce.	‘... *part of the challenge will be retaining family doctors especially female doctors if there isn’t more part time options ...’* (P8)	**6. Create flexible opportunities and ensure inclusivity and skill development for all** **:** no member of staff should be systematically excluded from training and development opportunities because they are part-time, have been off work for a period of time, female, less experienced, or hold a position perceived to be of lower importance to others.
A culture of medicine that prizes hard work and invincibility as a *‘* *badge of honour* (P3) exacerbates risk of burnout.	*‘There are so many societal expectations, colleague expectations, training expectations, financial expectations, that push us toward working harder, seeing more patients, and ignoring our own feelings.’* (P3)	**7. Create a supportive workplace ethos** **with a shared work ethic and regular contact with peers** **:** ensure all staff feel valued by patients, colleagues, and the organisation. Monitor working hours and create a culture where working overtime is seen as a problem that needs to be proactively addressed.
**At a systemic level**		
Legislative, licencing, and bureaucratic barriers can impact on females’ ability to undertake work.	*‘So there was no specific programme system that a doctor is trained in family medicine you either set up your own clinic or you worked in a private hospital with somebody there’s no organised healthcare system ...’* (P39)	**8. Knowledge of rights, laws, and best practice should be actively disseminated by national bodies** **:** ministries, training schemes, employers, medical licensing agencies, insurers, and professional member organisations can all play a part in ensuring that doctors are aware of the ‘rules’ that should protect their situations. It is especially important that these are shown to be applicable to the different settings where doctors may practice; small businesses, managed care organisations, and those that are self-employed need to be included, just as issues for females or LTFT workers may need specific information. Progressive and inclusive legislation and policymaking should routinely take a gender equity lens and make explicit the ways in which different situations may legitimately need different solutions within a framework of principles and values.
Expectation of full-time working for doctors in many countries can be a barrier to retention.	*‘I think the reality is that women have more family issues and especially in the Asian culture like we are expected to look after the kids and we take on that responsibility, and so should there be some kind of flexibility in the system.’* (P37)	**9. Careers and workforce planning should embrace and expect flexible working** **:** most doctors in most specialities will have periods where they need time out to be caregivers or for their own wellbeing; this is also relevant if people adopt more than one role or special interest. Professional and personal reasons for breaks in service and LTFT working should be treated equitably, and similar solutions may be found regardless of the reason. The culture and practice of medicine can be improved by career variation in a lifetime, and flexibility enhances both retention of talent and professional skills. Examples of good practice and innovative solutions should be shared at a systemic level (for example, via family medicine organisations).
Legally defined gender discrimination may be relatively rare, but experiences of gender inequity are common.	*‘Unfortunately we are not winning the battle yet … the requirements for women for a woman are much stricter than for a man and of course I experienced that personally where a man was chosen before me not because he had more qualifications’* (P17)	**10. Equitable career opportunities** **:** specific outreach is appropriate to ensure people access new opportunities and the monitoring of uptake by gender, speciality, and working background is appropriate.

LTFT = less than full-time. P = participant.

Box 1 **Categories of recommendations.**

**Knowledge:** help more family doctors to know about the literature on lifecycle transitions.

**Toolkits:** individual and team preparation can be used to address how best to support both individuals and their colleagues during transitions.

**Mentors:** create clear opportunities for support during and after lifecycle transitions.

**Systemic change:** careers and workforce planning should embrace and expect flexible working.

**Focus on gender:** addressing the underlying gender issues.

## Discussion

### ​Summary

The authors have found similarities in the experience of lifecycle transition events for female family doctors from across the world. Participants experienced competing demands, particularly relating to gendered expectations. Many women have the sense that the choice to be a doctor is a privilege and one should not expect to put personal needs first, yet there remains substantive expectations that females should be the lead caregivers in the home (and sometimes work) environment. All branches of medicine involve work where there is high demand and responsibility; acknowledging the need for balancing work and home demands and providing support in the workplace can give permission for female doctors to voice and meet their own needs. Conversely, spoken or unspoken assumptions that work comes first, inflexibility of hours and duties, and denial of personal differences in situations can lead to a lack of effective support, and in some cases a withdrawal from the workforce.

An important finding of the interviews and focus groups was that females’ resilience is not solely an internal construct or personal attribute, but depends on the availability of active support at every level. If this support is not available, they may be unable to sustain their professional career as intended. This research has identified a range of possible actions (Table 3) to address these barriers for female family doctors at the personal, and particularly team and systemic, level.

### ​Strengths and limitations

To the authors’ knowledge, this is the first study to explore female family doctors’ perceptions of factors affecting their resilience during lifecycle transitions. It benefits from having an international sample, allowing globally relevant lessons to be drawn. The use of purposive sampling to obtain a wide range of interview participants makes the present study findings more generalisable and enhances the credibility of the results. Methods such as interviewing until data saturation, member checking (participants checking the accuracy of interview transcripts), and authors interviewing as well as analysing the data also enhances trust in the findings.

Study limitations include the fact that interview participants were likely to be motivated to participate by personal experiences and a desire to help others in similar circumstances. As the study recruited only WONCA members, doctors who had stopped working may be under-represented (as they are less likely to retain their membership). The requirements for spoken English and access to Skype technology would have excluded some from study participation (and from understanding the advert); however, the range of experience and attitudes discussed, the participation of some doctors on a career break, and wide global representation suggests the sample was sufficiently heterogeneous to draw useful conclusions. Finally, as the authors only interviewed females, this study was unable to contrast comparable male perceptions.

The researchers are female family doctors based in the UK and US, and all but one author are experienced qualitative researchers. This was a strength in enabling recruitment, adding insight, and building rapport with participants. It also required the researchers to consider their own assumptions; these were checked by conscious iteration and mutual challenge during the coding. Focus group discussions were facilitated by the same author, who is well known as a senior WONCA leader. This may have affected the dynamic of the focus groups, but participant comments suggest it only enriched the discussion.

### ​Comparison with existing literature

Individuals who plan ahead, seek help, stay calm, and retain a sense of purpose are displaying known characteristics of resilience.^9,23^ The literature on personal and organisational resilience suggests that positive coping strategies and skills can be learned, and that resilient individuals are less likely to leave work because of stress or burnout;^23^ however, it is also clear that negative situations can destroy resilience, and that factors outside of the personal must be addressed to avoid workforce losses. The importance of external factors is in keeping with Maslow’s theory that achieving self-actualisation requires basic needs to be fulfilled.^26^ Females without sufficient support to meet both work and family needs experienced challenges due to competing demands, which influenced their career trajectory. This is in keeping with a 2016 study of rural female family doctors in the US, which found that reduced or flexible hours, supportive relationships, and clear boundaries were all successful strategies to achieve a work–life balance.^27^

These findings also echo those of Bridges,^15^ that a lifecycle transition that is unplanned, unsupported, and in which the individual lacks control, is more difficult to manage than those who have access to preparation and support, and in which an individual retains control and autonomy. Furthermore, the importance placed on support (through work and/or home) resonates with theories of social kinship and social capital,^28^ where societal norms and perceived obligations between family and friends maximise supportive acts.

This study identified few instances of direct gender discrimination. This may have been because gender was so intrinsic to their experiences that participants did not tend to identify it as a reason for differences in their treatment, which was found also in a recent US study.^6^

### ​Implications for practice

The challenges facing female family doctors have been described, and there is a necessity to build consensus around solutions to support female family doctors in the workplace. Female family doctors need to be empowered to have choices during transition and to have available to them structured and relational support when it is needed. The authors have gone some way towards describing possible recommendations, but these need testing in the field. Solutions need to be available to both males and females, as both groups experience the negative impacts of gender-related challenges.
